# Aortic valve anatomy and outcomes after transcatheter aortic valve implantation in bicuspid aortic valves

**DOI:** 10.1016/j.dib.2018.01.020

**Published:** 2018-01-31

**Authors:** Chiara De Biase, Antonios Mastrokostopoulos, Raphael Philippart, Louis Marie Desroche, Stephanie Blanco, Kamel Rehal, Nicolas Dumonteil, Didier Tchetche

**Affiliations:** Clinique Pasteur, Groupe Cardiovasculaire Interventionel (GCVI), Toulouse, France

## Abstract

This original clinical research study id focused on description of baseline anatomy and outcomes after transcatheter aortic valve implantation (TAVI) in patients presenting with severe aortic stenosis (AS) and bicuspid aortic valve (BAV). We compared this BAV population with a population of patients with AS and tricuspid aortic valves after a propensity score matching developed by a multivariate logistic regression according to a non-parsimonious approach. Baseline anatomical characteristics were obtained by transthoracic echocardiography (TTE) and multi-sliced computed tomography (MSCT) and compared by chi-square and t-student tests. Outcomes were evaluated by correct fisher test at in hospital and 30 days follow-up. We found that BAV patients presents more complicated baseline anatomy as compared to patients with tricuspid valves. These anatomical features lead to higher procedural complications as the need for a second device implantation. However this does not translate into increase in mortality rate at 30 days follow-up but rather correlate to a lower device success rate.

**Specifications Table**

**Table:** 

Subject area	*Interventional Cardiology*
More specific subject area	*Percutaneous valve implantation*
Type of data	*Table, text file, figure*
How data was acquired	*Statistical Package for the Social Sciences version 21.0 (SPSS v21.0, SPSS Inc, Chicago, IL). Student’s T test. Chi-square test.*
Data format	*Continuous variables are presented as mean ± standard deviation. Categorical variables are presented as count and percentages.*
Experimental factors	*None*
Experimental features	*None*
Data source location	*City: Toulouse, country: France.*
Data accessibility	*The data are available with this article*
Related research article	*Yoon SH, Bleiziffer S, De Backer O, Delgado V, Arai T, Ziegelmueller J, et al. Outcomes in Transcatheter Aortic Valve Replacement for Bicuspid Versus Tricuspid Aortic Valve Stenosis. Journal of the American College of Cardiology. 2017;69(21):2579-89. Epub 2017/03/24.*

**Value of the data**

• Aortic stenosis in bicuspid aortic valve (BAV) remains a challenge for transcatheter aortic valve implantation (TAVI).
• BAV patients presenting severe aortic stenosis are increasing in clinical practice
• There is little evidence concerning TAVI in this population.
• Our data can be an additional evidence for the feasibility of TAVI in BAV.
• Indeed many questions are still open to optimize the sizing and find some dedicated devices for this population. Registries and sizing comparisons between operators could find a way to improve TAVI procedures in this subset.

## 1 Data

– This study is a comparison analysis between patients with bicuspid or tricuspid aortic valves undergoing TAVI for severe AS.
– Patients with AS and BAV present more complicated baseline anatomy as compared to patients with tricuspid valves.
– These anatomical features correlate to a lower device success rate but are not related to increase in mortality rate at 30 days follow-up.
– The lower device success rate reported was mainly related to a second device implantation need since nor PVL or mean gradient and mortality rate were significantly different.
– Pre-procedural imaging and novel device technologies will help to address proper sizing and valve choice in the future.

## 2 Experimental design, materials, and methods

From January to December 2016, 460 patients with tricuspid aortic valve underwent TAVI procedure in our institution for symptomatic severe AS.

From January 2015 to April 2017, 83 consecutive patients with BAV had TAVI at our institution. The majority of these patients were indeed treated in 2016 (87%). Patients undergoing TAVI due to bio-prosthesis degeneration were not included.

**Fig. 1:**
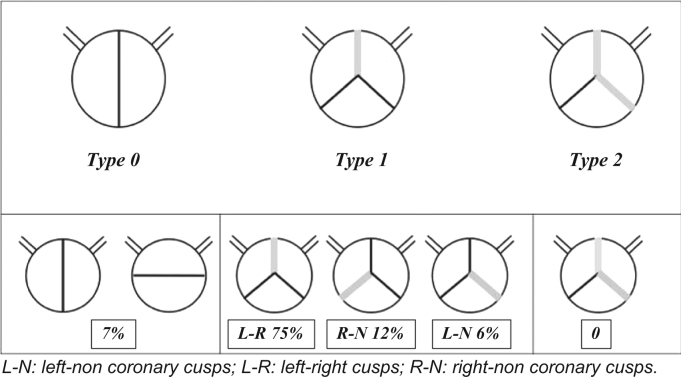
Bicuspid valve anatomy. L-N: left-non coronary cusps; L-R: left-right cusps; R-N: right-non coronary cusps.

BAV were classified following the Sievers classification as reported in Fig. 1 [1]. Multi-detector computed tomography (MDCT) and transthoracic echocardiogram (TTE) were assessed at baseline. BAV anatomy was identified by baseline MDCT after analysis using the 3mensio Structural Heart software version 8.0 (Pie Medical Imaging, Maastricht, the Netherlands) Workstation software. MDCT was the method of choice for sizing using the perimeter-derived diameter of the aortic annulus. In the BAV group, we used as additional measurement for sizing the inter-commissural distance 4 mm above the annulus.

Continue variables were compared using a Student’s *T* test and categorical variables with a chi-square test. A propensity-score matching was applied to account for differences in baseline characteristics of both groups.

A 1:2 propensity-score matching was performed on the basis of clinical risk factors for cardiovascular mortality and was developed by a multivariate logistic regression according to a non-parsimonious approach [2], [3]. A total amount of 249 patients, 83 with bicuspid and 166 with tricuspid aortic valve, were included in the final analysis.

**Fig. 2:**
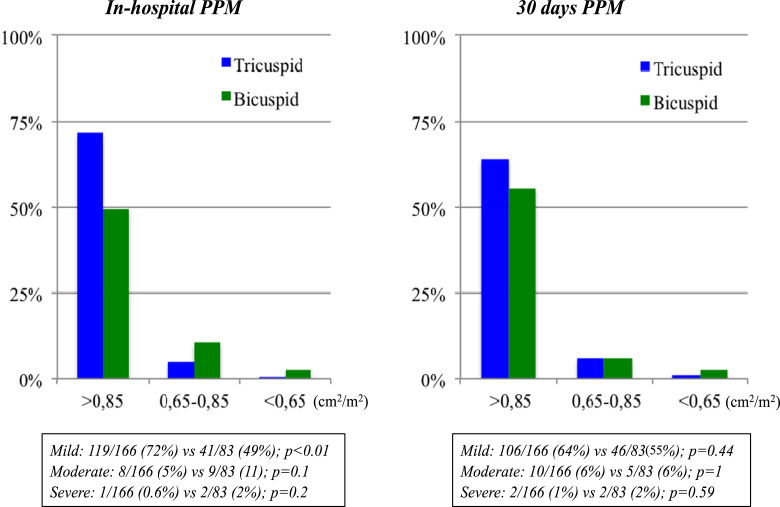
In-hospital and 30 days Prosthesis-patient mismatch (PPM).

Baseline anatomical features and procedural characteristics were compared between the two groups as previously described. Outcomes were evaluated following the Valve Academic Research Consortium-2 definitions (VARC)-2 definitions. PPM values were classified into mild, moderate, severe and were analyzed at both in-hospital and 30 days follow-up [4], [5], [6], [7] as reported in Fig. 2. Device success was evaluated at in-hospital follow-up and the early safety at 30 days [8].

The primary endpoint was all-cause mortality and early safety at 30 days. Secondary endpoint included device success.

Statistical significance was considered as *p* Value ≤ 0.05. All results were obtained using the Statistical Package for the Social Sciences version 21.0 (SPSS v21.0, SPSS Inc, Chicago, IL).
