# Supplementary material for: Aortic valve anatomy and outcomes after transcatheter aortic valve implantation in bicuspid aortic valves
Source: Data Brief. 2018 Jan 31;17:667–70. doi: 10.1016/j.dib.2018.01.020 (PMC5852276; doi:10.1016/j.dib.2018.01.020)
Supplement: Supplementary file 1 — Supplementary material [file mmc1.doc]

**Conflict of interest**

Toulouse 09/01/2018

Dear Editor of Data in Brief,

All the Authors have read and approved the manuscript entitled ” Aortic valve anatomy and outcomes after Transcatheter Aortic Valve Implantation in bicuspid aortic valves” which has never been send to another journal for the evaluation for publication.

These authors have the following conflicts of interest:

- Didier TCHETCHE is proctor for Edwards LifeSciences, Medtronic and Boston Scientific.
- Nicolas DUMONTEIL is proctor for Edwards LifeSciences, Medtronic and Boston Scientific.

We hope that you and your reviewers will find now the manuscript suitable for publication.

Best regards,

Dr. Chiara De Biase

chiadebiase@gmail.com

Clinique Pasteur GCVI

45 Avenue de Lombez

BP27617

31076 TOULOUSE CEDEX 3

FRANCE
